# Molecular detection of respiratory pathogens in tonsillar tissue from asymptomatic children: implications for result interpretation among the asymptomatic and symptomatic

**DOI:** 10.1128/spectrum.02872-25

**Published:** 2025-12-03

**Authors:** Amy L. Leber, Li Xu, Sophonie J. Oyeniran, Huanyu Wang

**Affiliations:** 1 Department of Pathology and Laboratory Medicine, Nationwide Children’s Hospital, Columbus, Ohio, USA; 2 Department of Pediatrics, Division of Infectious Diseases, Nationwide Children’s Hospital, The Ohio State University 2647, Columbus, Ohio, USA; 3 Department of Pathology, The Ohio State University 2647, Columbus, Ohio, USA; University of Maryland School of Medicine, Baltimore, Maryland, USA

**Keywords:** molecular detection, respiratory pathogens, tonsils

## Abstract

The diagnosis of upper respiratory infections is commonly made using nucleic acid amplification technologies (NAAT). The impact of asymptomatic carriage is not often appreciated. We aimed to detect respiratory pathogens present in tonsil tissues from children without acute infection. Remnant tonsil tissues were obtained from children undergoing tonsillectomy procedures. Nucleic acids of respiratory pathogens were detected using laboratory-developed PCR assays. The most frequently detected virus was Enterovirus, followed by Adenovirus and Rhinovirus. Among 522 enrolled children, 41.0% had at least one pathogen detected, and co-detections with more than one pathogen were found in 5.6%. Younger children were more likely to have a virus detected, and co-detection was more frequent. Results of standard of care testing for children with suspected respiratory infection during the same study period were also analyzed. Our data demonstrate that a wide array of pathogens can be detected in the tonsil tissues from children absent of acute respiratory symptoms. It emphasizes the need for careful interpretation of test results, particularly in the era of widespread multiplex NAAT usage.

**IMPORTANCE:**

The diagnosis of upper respiratory infections is now commonly made using nucleic acid amplification technologies (NAATs). One potential limitation of NAATs is the increased detection of the nucleic acids of pathogens in healthy individuals in the absence of symptoms. The findings from this study highlight the critical importance of understanding asymptomatic carriage of respiratory pathogens, particularly in pediatric populations. The detection of pathogens in asymptomatic individuals, especially younger children, calls for cautious interpretation of test results to avoid misdiagnosis and unnecessary treatment. It emphasizes the need for careful interpretation of test results, particularly in the era of widespread multiplex NAAT usage.

## INTRODUCTION

Human tonsils and adenoids are lymphoid epithelial tissues of the oral mucosa located between the oral cavity and the laryngopharynx at the gateway of the respiratory tract. The tonsillar epithelium provides spaces for microbiota colonization. Previous studies have demonstrated high detection rates of respiratory viruses, namely, Adenovirus (AdV), Rhinovirus (RV), and Enterovirus (EV), in tonsils or adenoids removed from children absent of acute respiratory infection (1). It is known that adenovirus can establish a persistent or latent infection in tissues such as the lung epithelial cells, adenoids, and tonsils. Species C adenoviruses, in particular, can establish persistent infections characterized by intermittent excretion (2).

The diagnosis of upper respiratory infections is now commonly made using nucleic acid amplification technologies (NAATs). One potential limitation of NAATs is the increased detection of the nucleic acids of pathogens in healthy individuals in the absence of symptoms. Also, in the era of multiplex molecular panels for the detection of respiratory tract pathogens, co-detection of multiple pathogens is becoming more and more common. It may be difficult to determine which pathogen is causing disease, and this is further confounded by the fact that many of these pathogens colonize the upper respiratory tract.

In this study, we examined the detection of pathogenic organisms in the upper respiratory tract in tonsillar tissue from children who underwent elective tonsillectomy and/or adenoidectomy (T&A) using NAATs to determine the extent of carriage. These data were compared to the detection of pathogens in symptomatic patients tested by a multiplex respiratory testing during the same time period.

## MATERIALS AND METHODS

### Study population

Children ≤21 years of age undergoing elective T&A were considered for enrollment. The criteria for enrollment included children who had to be without acute respiratory symptoms at the time of the operation and had not received antibiotics within 15 days prior. Patients were categorized according to the following age ranges: <3 years (group 1), 3 to <6 years (group 2), 6 to <9 years (group 3), and ≥9.0 years (group 4). Seasons were categorized as follows: spring (March–May), summer (June–August), fall (September–November), and winter (December–February).

### Total nucleic acid extraction and respiratory viruses and bacteria detection from tonsil tissue

Tonsillar tissue was homogenized with 400 µL of lysis buffer using a manual electricity-based homogenizer. Nucleic acids were extracted using the NucliSENS easyMag platform (bioMerieux, Durham, NC). A total of 400 µL of homogenate was extracted into 100 µL of elution solution.

Nucleic acids of respiratory pathogens were detected using laboratory-developed PCR assays (LDT-PCRs) targeting *Mycoplasma pneumoniae* (MP) (3), AdV (4), human EV, human RV (5), influenza A, influenza B, respiratory syncytial virus (RSV), parainfluenza virus 1–3 (PIV1–3), and human metapneumovirus (hMPV) (6). The sequences of the primers/probes are listed in Table 1.

**TABLE 1:** Primers and probes for detecting respiratory pathogens

Target	Gene target	Oligonucleotide	Sequence	Reference
Human AdV	Hexon gene	AQ1	5′-GCCACGGTGGGGTTTCTAAACTT-3′	4
		AQ2	5′-GCCCCAGTGGTCTTACATGCACATC-3′	
		AQ-P	5′-FAM-TGCACCAGACCCGGGCTCAGGTACTCCGA-TAMRA-3′	
Human EV	5′-noncoding region	ev2b	5′-GGCCCCTGAATGCGGCTAAT-3′	N/A^*a*^
		ev1b	5′-CAATTGTCACCATAAGCAGCCA-3′	
		evmgb	5′-FAM-CTTTGGGTGTCCGTGTT-MGB-3′	
Human RV	5′-noncoding region	Forward	5′-CPXGCCZGCGTGGC-3′	5
		Reverse	5′-GAAACACGGACACCCAAAGTA-3′	
		Probe	5′-FAM-TCCTCCGGCCCCTGAATGYGGC-BHQ1-3′	
Influenza A	M2 gene	FluA F	5′-GGACTGCAGCGTAGACGCTT −3’	N/A
		FluA R1	5’- CATCCTGTTGTATATGAGKCCCAT −3’	
		FluA R2	5’- CATYCTRTTGTATATGAGGCCCAT −3’	
		FluA P1	5′-VIC- CTCAGTTATTCTGCTGGTGCA -MGB-3′	
		FluA P2	5′-VIC- CTAAGCTATTCAACTGGTGCAC -MGB-3′	
Influenza B	HA gene	FluB F	5’- AATACGGTGGATTAAACAAAAGCAA −3’	N/A
		FluB R	5’- CCAGCAATAGCWCCGAAGAAA −3’	
		FluB *P*	5′-FAM -CATATTGGGCAATTTCCTA -MGB-3′	
RSV	Fusion gene	RSVA F	5′- GTAAGCAGCTCCGTTATCACATCTC −3′	N/A
		RSVA R	5′- TATTGGATGCTGTACATTTRGTTTTGC −3′	
		RSVA P	5′-VIC - AGGAGCCATTGTGTCATG -MGB-3′	
		RSVB F	5’- TCAATAAGCAAGAAGAGGAAACGA −3’	
		RSVB R	5′- ACTTGCTATTGCAGATCCTACACCTA −3′	
		RSVB P	5′-FAM - ATTTCTGGGCTTCTTG -MGB-3	
Parainfluenza virus 1	HN gene	PF1-1 F	5′-TTGGTCTACAACCCGAAATGAYAA-3′	N/A
		PF1-1 R	5′-CCTTCCTGCTGGTGTRTTAATG-3′	
		PF1-1 P	FAM-5′-TCCACGGTAAAYACAC-3′-MGB	
Parainfluenza virus 2		PF2-4	5′-GATGGAATCAATCGCAAAAGC-3′	
		PF2-5	5′-GCTACATAGCARTAYAAGAYACAACCT-3′	
		PF2-6	FAM-5′-TTCAGTCACTGCTATACC-3′-MGB	
Parainfluenza virus 3		PF3-7	5′-TGGCAYAGCAARTTACAATTAGGAA-3′	
		PF3-8	5′-GCACATTATGCCATGTCCATTT −3′	
		PF3-9	VIC-5′- ACTGAYTACAGTGATATAAG −3′-MGB	
hMPV	Nucleocapsid protein gene	NL-N-forward	5′-CATATAAGCATGCTATATTAAAAGAGTCTC-3′	6
		NL-N-reverse	5′-CCTATTTCTGCAGCATATTTGTAATCAG-3′	
		NL-N-probe	5′-FAM-TGYAATGATGAGGGTGTCACTGCGGTTG-TAMRA-3′	
MP	Adhesin P1 gene	MpnP1-for	5′-CCAACCAAACAACAACGTTCA-3′	3
		MpnP1-rev	5′-ACCTTGACTGGAGGCCGTTA-3′	
		MpnP1-probe	5′-VIC-TCAACTCGAATAACGGTGACTTCTTACCACTG-TAMRA-3′	

^*a*^ N/A, not applicable.

Thermocycling occurred using the ABI 7500 thermocycler (Life Technologies, Grand Island, NY) with the following running conditions: (i) RNA viruses: 25°C for 2 min, then 50°C for 15 min, followed by 10 min at 95°C and 45 cycles of 95°C for 15 s and 65°C for 1 min; (ii) MP and Adenovirus: 50°C for 2 min, denaturation at 95°C for 10 min and 45 cycles of 95°C for 15 s and 60°C for 1 min. The RV-PCR was designed to detect RV; however, it can occasionally cross-react with EV. In samples that tested positive for both EV and RV, the target with a lower cycle threshold (Ct) value was considered the true detection.

### Multiplex testing for viruses among symptomatic children

Results of standard of care testing for children with suspected respiratory infection during the same study period were analyzed. The test used was the BioFire Respiratory Panel v2.0 (BioFire Diagnostics, Salt Lake City, UT, USA), which detects AdV, Coronaviruses, hMPV, RV/EV, FluA, FluB, PIV1-4, RSV, MP, *Bordetella pertussis*, *Bordetella parapertussis*, *Chlamydia pneumoniae*, and MP. The sample tested was a nasopharyngeal (NP) swab placed in transport media. This test does not differentiate RV and EV; therefore, the detection of RV and/or EV is reported as RV/EV. The detection rates of AdV, RV/EV, FluA, FluB, RSV, hMPV, and MP were analyzed. The BioFire Respiratory panel and all the LDT-PCRs used in this study were fully validated for clinical use and comparative studies as requested by the College of American Pathologists (COM.04250) (7) showed they had similar performance across all targets.

### Demographic and clinical data collection

Demographics, including age, gender, and post-surgical diagnosis, were collected from the electronic health-care record for the enrolled patients.

### Statistical analysis

Continuous variables (i.e., patient’s age) were analyzed using the Kruskal-Wallis test, and data were reported as medians and 25%–75% interquartile ranges (IQR). Categorical variables were analyzed by χ^2^ and *P* < 0.05 was considered significant. The analysis was conducted using GraphPad Software (GraphPad Software, San Diego, CA).

## RESULTS

### Patient information

From April 2018 to January 2020, tonsillar tissues were collected from 522 individual patients who underwent an elective T&A at Nationwide Children’s Hospital. The median age was 4.6 years (IQR: 3.3, 6.5 years), and 279 (53.5%) were male. The indications for the surgery were determined by their primary care physicians in conjunction with ear, nose, and throat specialists. At the time of surgery, none had symptoms of acute respiratory infection, nor did they receive antibiotics within 15 days prior. Four hundred and ninety-nine (95.6%) children had adenotonsillar hypertrophy, and 84 (16.9%) had recurrent or chronic tonsillitis with or without adenotonsillar hypertrophy. Ninety-two (17.6%) were children <3 years (group 1); 274 (52.5%) were 3 to <6 years (group 2); 95 (18.2%) were 6 to <9 years (group 3); and 61 (11.7%) were ≥9 years (group 4).

### Respiratory pathogen detection rates in tonsils from asymptomatic children

**Fig 1:**
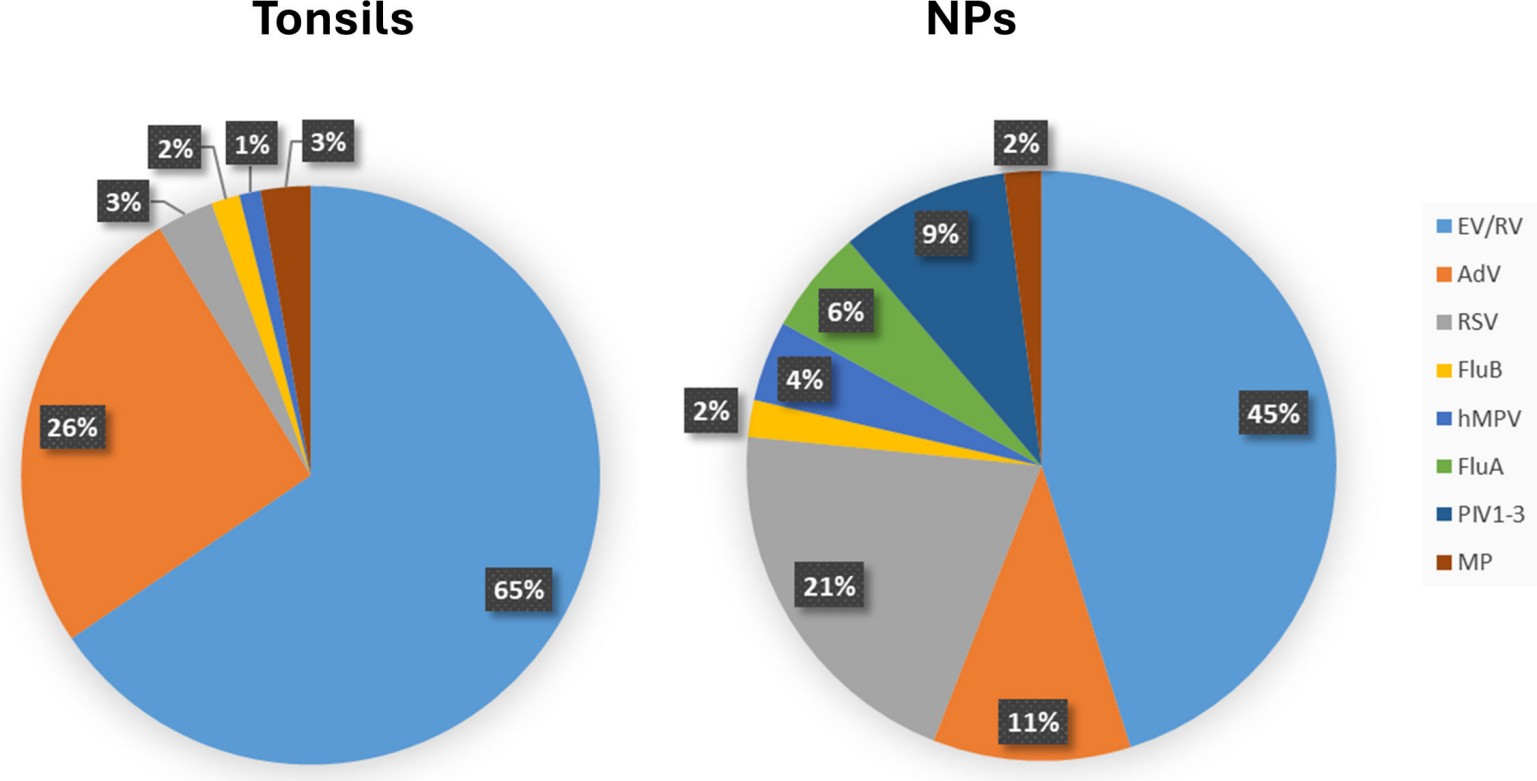
Detection of respiratory pathogens in tonsils from asymptomatic children and NPs from symptomatic children between April 2018 and January 2020.

The detection rates of each respiratory pathogen in tonsils are presented in Table 2 and Fig. 1. Among asymptomatic children, the most frequently detected virus was EV (26.3%), followed by AdV (12.5%) and RV (5.4%). FluA and PIV1-3 were not detected. All other pathogens were detected in frequencies less than 5%. Two hundred and fourteen children (41.0%) had at least one pathogen detected, and co-detection with more than one respiratory pathogen was found in 29 (5.6%) children; 24 (82.8%) out of these 29 had EV detected.

**TABLE 2:** Detection rates of respiratory pathogens in tonsils from asymptomatic children by age groups^*a*^

	Total cohort	Group 1	Group 2	Group 3	Group 4	*P* value
Age groups		<3 years	3 to <6 years	6 to <9 years	≥9 years	
Number	522	92	274	95	61	
Gender, M (%)	279 (53.5)	53 (57.6)	152 (55.5)	45 (47.4)	29 (47.5)	
Respiratory pathogens, *n* (%)						
EV	137 (26.3)	40 (43.5)	71 (25.9)	18 (19.0)	8 (13.1)	<0.0001
AdV	65 (12.5)	17 (18.5)	39 (14.2)	3 (3.2)	6 (9.8)	0.008
RV	28 (5.4)	5 (5.4)	17 (6.2)	4 (4.2)	2 (3.3)	NS^*b*^
RSV	8 (1.5)	3 (3.3)	5 (1.8)	0	0	NS
FluB	4 (0.8)	1 (1.1)	1 (0.4)	1 (1.1)	1 (1.6)	NS
MPV	3 (0.6)	2 (2.2)	0	1 (1.1)	0	NS
FluA	0	0	0	0	0	NS
PIV 1–3	0	0	0	0	0	NS
MP	7 (1.3)	0	5 (1.8)	2 (2.1)	0	NS
Detection, *n* (%)						
0	308 (59.0)	39 (42.4)	155 (56.6)	70 (73.7)	44 (72.1)	<0.0001
≥1	214 (41.0)	53 (57.6)	119 (43.4)	25 (26.3)	17 (27.9)	<0.0001
≥2	29 (5.6)	13 (14.1)	13 (4.7)	3 (3.2)	0	0.0005
EV among viral co-detection, *n* (%)	24 (82.8)	13 (100)	8 (61.5)	3 (100)	NA^*c*^	NA

^*a*^ PIV, parainfluenza virus.

^*b*^ NS, not significant.

^*c*^ NA, not available.

Younger children (groups 1 and 2) were more likely to have a virus detected, and the co-detection with more than one virus was also more frequent (*P* < 0.05, Table 2). Among 92 children younger than 3 years old, 13 (14.1%) had at least two viruses detected, and no children older than 9 years had co-detection. EV and AdV were most frequently detected in the youngest age group (group 1), and the detection rates for EV trended down as the age groups increased.

### Respiratory pathogen detection rates in NP from symptomatic children

During the same study period, 21,619 NP samples were tested as part of standard of care, and the detection rates for each pathogen were presented (Table 3; Fig. 1). The most frequently detected respiratory pathogen was EV/RV (35.8%), followed by RSV (16.4%), AdV (8.6%), and PIV1–3 (7.2%). The detection rates of other pathogens were lower, but all were greater than 1%. Co-detection was found in 16.2% of individuals, among which 72.4% of individuals had EV/RV detected.

**TABLE 3:** Detection rates of respiratory pathogens in NP from symptomatic children by age groups^*a*^

	Total cohort	Group 1	Group 2	Group 3	Group 4	*P* value
Age groups		<3 years	3 to < 6 years	6 to < 9 years	≥9 years	
Number	21,619	12,536	3,093	1,872	4,118	
Gender, M (%)						
Respiratory pathogens, *n* (%)						
EV/RV	7,744 (35.8)	5,059 (40.3)	1,235 (39.9)	605 (32.3)	845 (20.5)	<0.0001
AdV	1,861 (8.6)	1,277 (10.2)	326 (10.5)	138 (7.4)	120 (2.9)	<0.0001
RSV	3,539 (16.4)	2,854 (22.7)	458 (14.8)	117 (6.3)	110 (2.7)	<0.0001
FluB	346 (1.6)	94 (0.7)	80 (2.6)	83 (4.4)	89 (2.2)	<0.0001
MPV	759 (3.5)	501 (4.0)	154 (5.0)	47 (2.5)	57 (1.4)	<0.0001
FluA	978 (4.5)	331 (2.6)	188 (6.1)	198 (10.6)	261 (6.3)	<0.0001
PIV 1–3	1,589 (7.2)	1,042 (8.3)	270 (8.7)	110 (5.9)	104 (2.5)	<0.0001
MP	348 (1.6)	66 (0.5)	63 (2.0)	92 (4.9)	127 (3.1)	<0.0001
Viral detection, *n* (%)						
0	7,015 (32.4)	2,038 (16.2)	727 (23.5)	613 (32.7)	2,437 (59.2)	<0.0001
≥1	14,604 (67.6)	9,315 (83.8)	2,366 (76.5)	1,259 (67.3)	1,681 (40.8)	<0.0001
≥2	3,505 (16.2)	2,353 (18.7)	558 (18.0)	235 (12.6)	177 (2.8)	<0.0001
EV/RV among viral co-detection, *n* (%)	2,538 (72.4)	1,865 (79.3)	412 (73.8)	152 (64.7)	109 (61.6)	<0.0001

^*a*^ PIV, parainfluenza virus.

EV/RV, AdV, RSV, and PIV1–3 were more frequently detected in younger children (groups 1 and 2, Table 3), and the detection rates trended down as the age groups increased. Co-detection was also more frequent in younger children with EV/RV being the most common co-detected target. The highest detection rates for FluA, FluB, and MP were found with group 3 (6 to <9 years).

### Respiratory pathogen detection rates among symptomatic vs asymptomatic children

Both LDT-PCRs and BioFire Respiratory Panel were fully validated for clinical use in our institution. Our quality assurance activities and assay comparison studies have consistently shown that the performance of the LDT-PCRs is comparable to that of the BioFire panel for the respiratory pathogens evaluated in this study. Therefore, we compared the detection rates of these pathogens in tonsil samples from asymptomatic children and NP samples from asymptomatic children. As shown in Table 4, hMPV, FluA, PIV1–3, and RSV were significantly more prevalent in symptomatic individuals’ NP when compared to asymptomatic individuals’ tonsils (*P* < 0.05). AdV, on the contrary, was less prevalent in symptomatic individuals (*P* > 0.05). The detection rates of EV/RV, FluB, and MP were no different between the two groups.

**TABLE 4:** Detection rates of respiratory pathogens in tonsils from asymptomatic children and NPs from symptomatic children between April 2018 and January 2020^*b*^

	Asymptomatic	Symptomatic	*P* value
Number	522	21,619	
Respiratory pathogens, *n* (%)			
EV/RV^*a*^	165 (31.6)	7,744 (35.8)	NS (0.052)^*c*^
AdV	65 (12.5)	1,861 (8.6)	0.0035
RSV	8 (1.5)	3,539 (16.4)	<0.0001
FluB	4 (0.8)	346 (1.6)	NS (0.1545)
hMPV	3 (0.6)	759 (3.5)	<0.0001
FluA	0	978 (4.5)	<0.0001
PIV 1–3	0	1,589 (7.2)	<0.0001
MP	7 (1.3)	348 (1.6)	NS (0.8589)
Detection, *n* (%)			
0	308 (59.0)	7015 (32.4)	<0.0001
≥1	214 (41.0)	14,604 (67.6)	<0.0001
≥2	29 (5.6)	3,505 (16.2)	<0.0001
EV/RV among viral co-detection, *n* (%)	24 (82.8)	2,538 (72.4)	NS (0.2631)

^*a*^ Comparison to the combined targets EV and RV due to the cross-reactivity with the test used for symptomatic patients.

^*b*^ PIV, parainfluenza virus.

^*c*^ NS, not significant.

### Distribution of AdV, EV, and RV according to seasons from asymptomatic and symptomatic children

**Fig 2:**
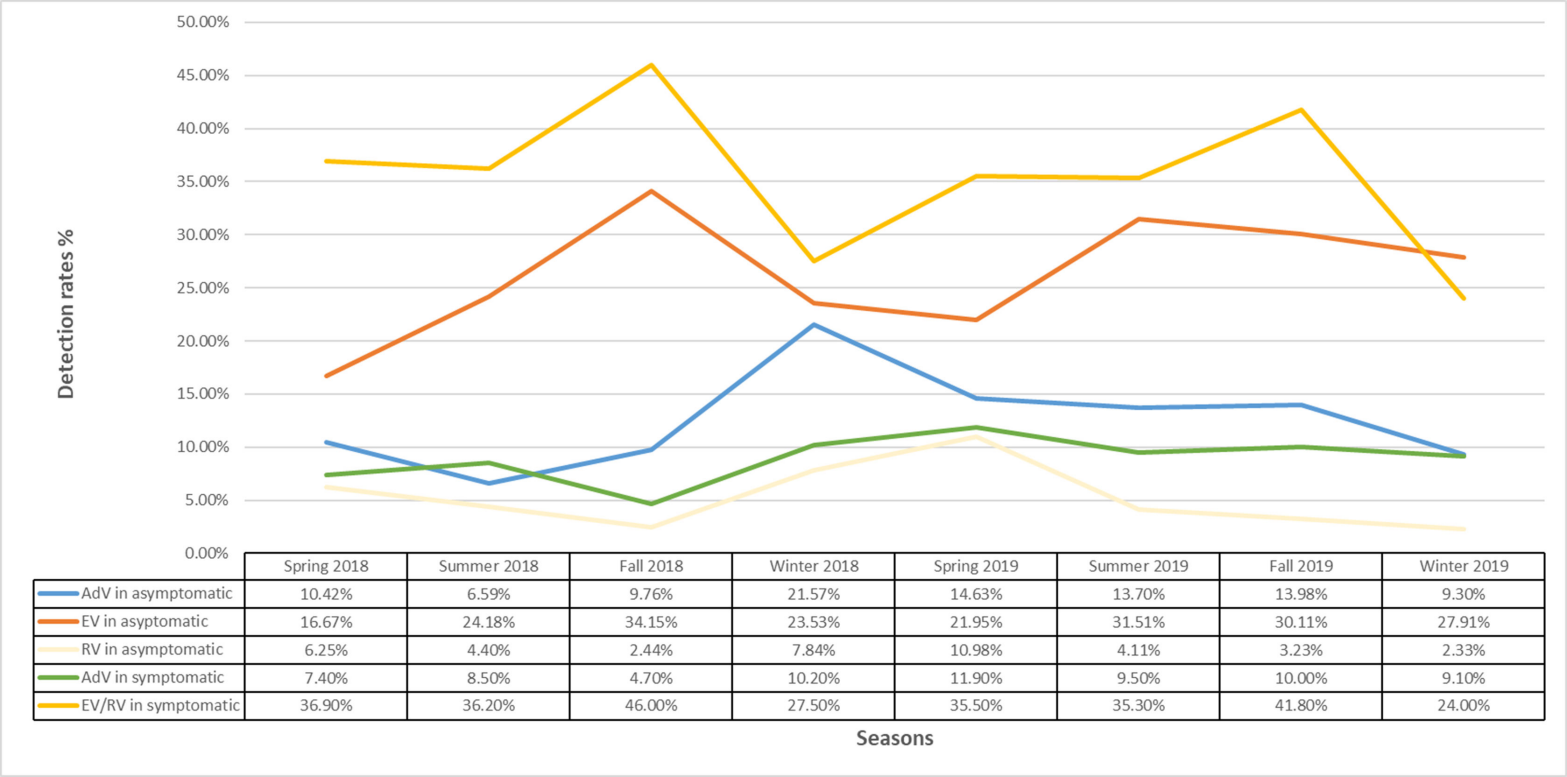
Distribution of AdV, EV, and RV according to seasons from asymptomatic and symptomatic children.

The detection rates of AdV, EV, and RV in tonsils from asymptomatic children fluctuated throughout the years, and there was no significant viral seasonality. Higher detection was seen for AdV in winter and spring, RV in summer and fall, and EV in spring (Fig. 2). Other pathogens were not evaluated for their seasonality as the prevalence was too low. The detection of AdV and EV/RV in NP from symptomatic children by the multiplex molecular panel also fluctuated: higher activity was seen for AdV in winter and spring, and EV/RV in fall.

## DISCUSSION

The present study revealed high detection rates of respiratory viruses in tonsillar tissue in asymptomatic children who underwent T&A. Detection of respiratory viruses in the adenoids or tonsillar tissues from children absent of acute respiratory symptoms has been reported (8–12). EV, RV, and AdV were commonly detected with high prevalence, and other respiratory viruses (FluA, FluB, HMPV, PIV, and RSV) were less likely to be detected. The detection rates of these viruses varied by study population and geographic location and were also impacted by seasonal effect, sample size, and detection methods. In our study that spanned 2 years and included 522 children, 41% of tonsillar tissues had at least one virus detected, and 5.9%, 26.3%, and 12.5% samples were positive for RV, EV, and AdV, respectively. Proenca-Modena et al. reported that detection of respiratory viruses in 121 children who underwent T&A varied among sample sites (nasopharyngeal washes (NS), palatine tonsils, and adenoids), indicating that they may have different tissue tropisms (9): RV had the highest prevalence in NS (42.7%), followed by AdV (29.8%) and EV (18.2%); while in tonsils sample, EV was found highest (37.2%), followed by AdV (24%) and RV (12.4%). These viral prevalence rates in tonsil samples are consistent with our findings. Proenca-Modena et al. also found among all patients who had samples collected simultaneously from NS, tonsils, and adenoids, half (49.6%) had virus detected in all three sample types, indicating that viruses that colonize tonsils or adenoids can also be detected from nasopharyngeal or nasal specimens which are the most common specimen types for molecular detection of respiratory viruses. In addition, studies focused on NP revealed that the RV and EV RNA were commonly detected throughout the year in the NP of children or young adults who did not have concurrent respiratory symptoms (13–15).

Molecular detection of respiratory pathogen is now the standard of care for the diagnosis of acute respiratory illness. Our findings and the aforementioned studies support the hypothesis that positive PCR results, particularly EV, RV, and AdV from upper respiratory specimen, should be carefully interpreted as they may represent long-lived viral shedding from a remote infection or carriage. It is worth noting that data from the same study period (April 2018 to January 2020) of the multiplex NAATs respiratory panel used for patient testing at our institution showed a 35.8% (Table 3) EV/RV positive rate in individuals tested due to concern for respiratory viral infection. Among 16.2% of the samples that had ≥2 pathogens detected, 72.4% had EV/RV co-detected. This test does not differentiate EV from RV. These high detection rates have also been reported elsewhere in the United States. Haddadin et al. found EV/RV to be the most commonly detected target with rates of 22.5% and 22.6% in 2018 and 2019, respectively, in a tertiary hospital using the same multiplex panel (16). Another multi-center outpatient pediatric study reported EV/RV detection rates ranging from 39.65% in 2018 to 27.2% in 2022, with EV/RV accounting for 73.2% and 72.2% of co-infections before and during the COVID-19 pandemic, respectively (17). In our cohort who were absent of respiratory symptoms, EV had a 31.6% prevalence, and 24 out of 29 (82.8%) samples with ≥2 virus detections were positive for EV. Furthermore, in both groups, EV/RV was detected with the highest rate in the youngest age group, and the detection rates trended down as the age increased (*P* < 0.05). This difference in the detection by age group has also been observed in other studies, which reported the highest EV/RV detection in young children compared with older children in a symptomatic cohort (18). Interestingly, the detection rates of AdV were higher in the asymptomatic group (12.5%) than in the symptomatic group (8.6%, *P* = 0.0035), and like EV/RV in both groups, the prevalence was higher in the younger children (Groups 1 and 2). This suggests that EV/RV and AdV are commonly detected in young children with and without upper respiratory tract symptoms; therefore, it could represent carriage rather than the cause of disease. In contrast, there were no detections for FluA and PIV and very low detection for FluB, PIV1-3, RSV, and hMPV in our asymptomatic cohort, but the detection rates of these viruses in symptomatic individuals by the multiplex panel were far higher. This suggests that detection of these viruses in symptomatic children is more likely to represent the cause of an infection than a carriage status.

Carriage rates of MP in asymptomatic individuals differ and range from 0% to 21% as summarized by Waites et al. (19). The detection rate was 1.3% in our cohort, while the detection rate in symptomatic children’s NP by the multiplex NAATs from the same study period was 1.1%. The low carriage rate in our cohort could be explained by the low prevalence in our population. More work is needed to differentiate the carriage status from infection.

One major limitation of this study is that although the children went into the surgery without acute respiratory symptoms, they did have conditions that warranted tonsillectomy, and they may not represent the general pediatric population. However, tonsillar tissue from healthy individuals for study is rarely available. The RV-PCR used in this study can cross-react with the EV-PCR. However, the rate of cross reaction is low; among 28 RV-positive tonsil samples, three (10.7%) were also positive by EV-PCR but with higher Ct values, thus allowing differentiation. In addition, this study was conducted in the pre-pandemic era, while a recent study discovered that SARS-CoV-2 could persist in the tonsillar tissue in children without symptoms of COVID-19 (20). More work is needed to understand the impact of the COVID-19 pandemic on the persistence and dynamics of respiratory viruses and bacteria in the upper respiratory tract in children. Studies extending into the post-pandemic period could provide valuable insights into how the pandemic-related public health interventions have influenced pathogen colonization and persistence as well as their potential impact on the interpretation of NAAT results.

In summary, our data demonstrate that a wide array of pathogens can be detected in the tonsil tissues from children absent of acute respiratory symptoms. Detecting the nucleic acids of respiratory viruses like RV, EV, or AdV by NAATs in asymptomatic children requires cautious interpretation, as they may represent a carriage status not an infection. Therefore, testing asymptomatic children with a multiplex NAATs panel should be reserved for those with risk factors for severe outcomes. Detection of EV, RV, or AdV in symptomatic patients may also represent carriage, especially in young children or when co-detected with other pathogens. This study supports the utility of diagnostic stewardship to prevent unnecessary testing, which could complicate the result interpretation without patient benefit. It emphasizes the need for careful interpretation of test results, particularly in the era of widespread multiplex NAAT usage.
